# Accidental administration of the remifentanil formulation Ultiva™ into the epidural space and the complete time course of its consequences: a case report

**DOI:** 10.1186/s40981-016-0046-5

**Published:** 2016-08-08

**Authors:** Kota Nishimoto, Sachiyo Sakamoto, Makiko Mikami, Kiichi Hirota, Koh Shingu

**Affiliations:** Department of Anesthesiology, Kansai Medical University, 2-3-1 Shinmachi, Hirakata, 573-1191 Japan

**Keywords:** Ultiva™, Remifentanil, Glycine, Epidural space, Accidental administration

## Abstract

**Background:**

Ultiva™ contains the potent short-acting μ-opioid receptor agonist remifentanil hydrochloride, and it is commonly administered intravenously during general anesthesia. It is not approved for epidural or intrathecal use in clinical practice because it contains glycine as an acidic buffer. However, at this moment, very limited information is available on epidural administration of Ultiva™.

**Case presentation:**

We report the accidental administration of 300 μg of remifentanil and 2.25 mg of glycine into the epidural space after emergence from general anesthesia for distal pancreatectomy and the complete time course of its consequences. The respiratory depression occurred at 5 min after the administration, and complete loss of consciousness was observed at 8 min. The patient was re-intubated and underwent mechanical respiration. At 45 min (33 min after re-intubation), spontaneous respiration resumed, she was responsive to commands, and her orientation returned. She was extubated successfully.

**Conclusions:**

These consequences might have resulted from the diffusion of the components of Ultiva™ into not only systemic circulation but also the cerebrospinal fluid. Moreover, the complex pathophysiology might be associated with remifentanil, as well as glycine present in Ultiva™.

## Background

Ultiva™ is commonly administered intravenously during general anesthesia, and the principal ingredient is the potent short-acting μ-opioid receptor agonist remifentanil hydrochloride. Studies in rats have suggested that intrathecal administration of remifentanil can induce profound analgesia [1, 2]. Ultiva™ is not approved for epidural or intrathecal use in clinical practice because it contains glycine as an acidic buffer. Thus, very limited information is available on epidural administration of Ultiva™. Here, we report the accidental administration of 300 μg of remifentanil and 2.25 mg of glycine into the epidural space after emergence from general anesthesia for distal pancreatectomy and the complete time course of its consequences.

## Case presentation

A 67-year-old woman (height, 149 cm; weight, 57 kg) with pancreatic insulinoma was admitted for laparoscopic tumor resection. She claimed experiencing intermittent hypoglycemic attacks due to insulinoma, and therefore, she was prescribed diazoxide. At the preoperative consultation, she was classified as American Society of Anesthesiologists Physical Status II, and general anesthesia supplemented by epidural block was scheduled.

Her preoperative blood pressure was 150/90 mmHg, and her heart rate was 95 beats/min in the supine position in the operating room. After placement of the epidural catheter at the Th8–Th9 epidural space, general anesthesia was induced with propofol (50 mg) and fentanyl (100 μg). Intubation of the trachea was facilitated with rocuronium. Anesthesia was maintained with 1.25–1.5 % sevoflurane in air/oxygen and 0.05–0.1 μ/kg/min remifentanil. Additionally, levobupivacaine (0.25 %) was intermittently administered into the epidural space. The surgery lasted for 5 h and 7 min and was uneventful. The estimated blood loss was 377 mL, and a total of 3900 mL of crystalloid fluid was infused during the perioperative period. The urine output was 1880 mL. The patient emerged from anesthesia promptly and was successfully extubated. After extubation, she was alert and fully responsible. Her respiratory rate was 16 breaths/min, and her SpO_2_ level was 100 % under 3 L/min O_2_ insufflation. However, she complained of abdominal pain, and 3 mL of a solution, which was believed to be 0.25 % levobupivacaine, was administered into the epidural space. At 3 min after administration, her pain subsided; however, the SpO_2_ level decreased to 95 %. At this time point, she was alert and responsible and could take a deep breath in response to a command. At 5 min, the SpO_2_ level decreased to 90 %, and her conscious level reduced to Japan Coma Scale (JCS) 30. At this time point, it was found that the solution administered was 3 mL of Ultiva™ (100 μg/mL) dissolved in 0.9 % NaCl. At 8 min, her SpO_2_ level was 93 % and conscious level was JCS200. She underwent mask ventilation for 5 min; however, the consciousness level did not improve. At 13 min after administration, her trachea was intubated, with 100 mg of propofol. A muscle relaxant was not used. Because spontaneous respiration was not detected after intubation, she underwent mechanical ventilation. At 45 min (33 min after re-intubation), spontaneous respiration resumed, she was responsive to commands, and her orientation returned. She was extubated successfully and was transferred to the ward. On assessment, she did not demonstrate any neurological deficit.

### Discussion

We reported the accidental epidural administration of the potent opioid reagent Ultiva™ and the complete time course of its consequences.

In our case, the first symptom was a decrease in the SpO_2_ level, which was detected 3 min after the epidural administration of Ultiva™ solution. Intriguingly, at 10 min, spontaneous respiration was preserved. According a pharmacokinetics model [3, 4], after intravenous bolus administration of 300 μg of remifentanil, the peak whole blood concentration of remifentanil rapidly reached 33 ng/mL and then the concentration decreased rapidly to 1.53 ng/mL within 15 min and after 30 min the concentration decreased to 0.058 ng/ml. In contrast, the effect site concentration peaked to 17.2 ng/mL and then decreased to 2.3 ng/mL within 15 min. Thus, the time course of our case differs from that of the pharmacokinetics model. The discrepancy may have been caused by a delay in the diffusion of remifentanil from the epidural space to systemic circulation or by diffusion of remifentanil from the epidural space to the cerebrospinal fluid. The diffusion of remifentanil from the epidural space to the cerebrospinal fluid is supported by the fact that respiratory suppression and consciousness disturbance continued for more than 40 min in our case. These consequences would have not occurred for such a long duration if the effects were systemic.

In the present case, we used a 2 mg formulation of Ultiva™, which contains 15 mg of glycine as an adjunct. Thus, the consequences might be partly due to the effects of glycine on the central nervous system, in addition to the effects of remifentanil. Glycine has been shown to be not only an inhibitory neurotransmitter but also an *N*-methyl-d-aspartate receptor coactivator [5, 6]. A previous study reported that intrathecal administration of Ultiva™ to substantia gelatinosa neurons hyperpolarized the membrane potentials and depressed presynaptic glutamate release predominantly through the activation of glycine receptors [7]. Moreover, intrathecal glycine was shown to cause reversible motor impairment in a rat model [1].

In this case, we did not use naloxone to antagonize the effect of remifentanil to alleviate the adverse phenomena based on the two reasons. We thought that if the disturbance of consciousness and loss of spontaneous respiration was due to the effect of remifentanil, the symptoms may disappear soon. However, intravenous administration of naloxone might be a causal treatment and have elucidated the etiology of the symptoms.

A previous report presented a case of epidural administration of 567 μg of remifentanil hydrochloride before induction of general anesthesia [8]. In that case, the patient became unconscious and developed rigidity of the chest and abdominal muscles. General anesthesia was introduced immediately after the administration, and therefore, the complete time course of the consequences was not demonstrated. In contrast, in our case, rigidity of the chest and abdominal muscles was not observed. In addition, our case demonstrated the full time course of respiratory suppression and consciousness disturbance. Therefore, to our knowledge, this is the first report of the complete time course of the consequences of epidural administration of Ultiva™.

The sole cause of the accident described in this case is certainly the wrong administration of Ultiva™ solution into the epidural space. The more intensive attention should have be paid to “five rights” concept including right patient, right drug, right dose, right route, and right time [9]. The differential usage of syringe (e.g., volume and color) for venous and epidural administration might have prevented the occurrence of the incident.

In the morning of postoperative day (POD) 1, the patient was fully alert and responsive. Neither sensory nor motor neurological disturbance was observed. The patient was discharged on foot on POD 15 without further complications.

## Conclusions

We reported a case of accidental administration of the remifentanil formulation Ultiva™ into the epidural space. Complete loss of consciousness and respiratory depression continued for 45 min. These consequences might have resulted from the diffusion of the components of Ultiva™ into not only systemic circulation but also the cerebrospinal fluid. Moreover, the complex pathophysiology might be associated with remifentanil, as well as glycine present in Ultiva™.

## References

[1] Buerkle H, Yaksh TL (1996). Continuous intrathecal administration of shortlasting mu opioids remifentanil and alfentanil in the rat. Anesthesiology.

[2] Buerkle H, Yaksh TL (1996). Comparison of the spinal actions of the mu-opioid remifentanil with alfentanil and morphine in the rat. Anesthesiology.

[3] Minto CF, Schnider TW, Egan TD, Youngs E, Lemmens HJ, Gambus PL (1997). Influence of age and gender on the pharmacokinetics and pharmacodynamics of remifentanil. I Model Dev Anesthesiol.

[4] Minto CF, Schnider TW, Shafer SL (1997). Pharmacokinetics and pharmacodynamics of remifentanil. II Model Appl Anesthesiol.

[5] Glass PS, Hardman D, Kamiyama Y, Quill TJ, Marton G, Donn KH (1993). Preliminary pharmacokinetics and pharmacodynamics of an ultra-short-acting opioid: remifentanil (GI87084B). Anesth Analg.

[6] Guntz E, Dumont H, Roussel C, Gall D, Dufrasne F, Cuvelier L (2005). Effects of remifentanil on N-methyl-D-aspartate receptor: an electrophysiologic study in rat spinal cord. Anesthesiology.

[7] Sumie M, Shiokawa H, Yamaura K, Karashima Y, Hoka S, Yoshimura M (2016). Direct Effect of Remifentanil and Glycine Contained in Ultiva(R) on Nociceptive Transmission in the Spinal Cord: In Vivo and Slice Patch Clamp Analyses. PLoS One.

[8] Xu X, She S, Yao S, Mok M, Zuo Z (2007). Respiratory depression and difficult ventilation after inadvertent epidural administration of remifentanil. Anesth Analg.

[9] Smeulers M, Verweij L, Maaskant JM, De Boer M, Krediet CT, Nieveen Van Dijkum EJ (2015). Quality indicators for safe medication preparation and administration: a systematic review. PLoS One.

